# Pro-inflammatory microbiota mediates the effect of host risk genes in Crohn’s disease

**DOI:** 10.3389/fmicb.2026.1856205

**Published:** 2026-06-03

**Authors:** Dawei Gong, Shanshan Zhang, Heng Zhu, Jicai Lv, Liguo Sun, Ziying Chen, Leisheng Zhang, Shumin Zhang

**Affiliations:** 1 Department of Gastroenterology and Endoscopy Center, The Fourth People's Hospital of Jinan, Jinan, China; 2 Shandong Provincial Key Medical and Health Laboratory of Blood Ecology and Biointelligence, Jinan Key Laboratory of Medical Cell Bioengineering, Science and Technology Innovation Center, The Fourth People’s Hospital of Jinan Affiliated to Shandong Second Medical University, Jinan, China; 3 Department of Quality Management, The Fourth People's Hospital of Jinan Affiliated to Shandong Second Medical University, Jinan, China; 4 Department of Hepatology II, Shandong Public Health Clinical Center, Shandong University, Jinan, China

**Keywords:** Crohn’s disease, genetics, genome-wide association studies, inflammation, microbiota

## Abstract

Crohn’s disease (CD) is a chronic inflammatory disease mainly characterized by chronic and relapsing intestinal inflammation. The pathogenesis of CD involves dysregulation of the immune response to commensal gut pro-inflammatory microbiota. Genome-wide association studies (GWAS) identified many genetic susceptibility loci associated with CD, such as *NOD2, CARD9, ATG16L1, IRGM*, and *FUT2*, etc. These risk genes are associated with the relative abundance of particular bacteria. The combined risk score was found to be predictive of disease severity. Moreover, the exact composition of microbiota is known to determine host phenotypes, including metabolic and immunological phenotypes. In this study, we elucidated the association between host genetics, gut microbiota, and host phenotypes. We propose that the pathogenesis of Crohn’s disease is mediated by pro-inflammatory microbiota in genetically susceptible individuals.

## 1 Introduction

**Figure 1:**
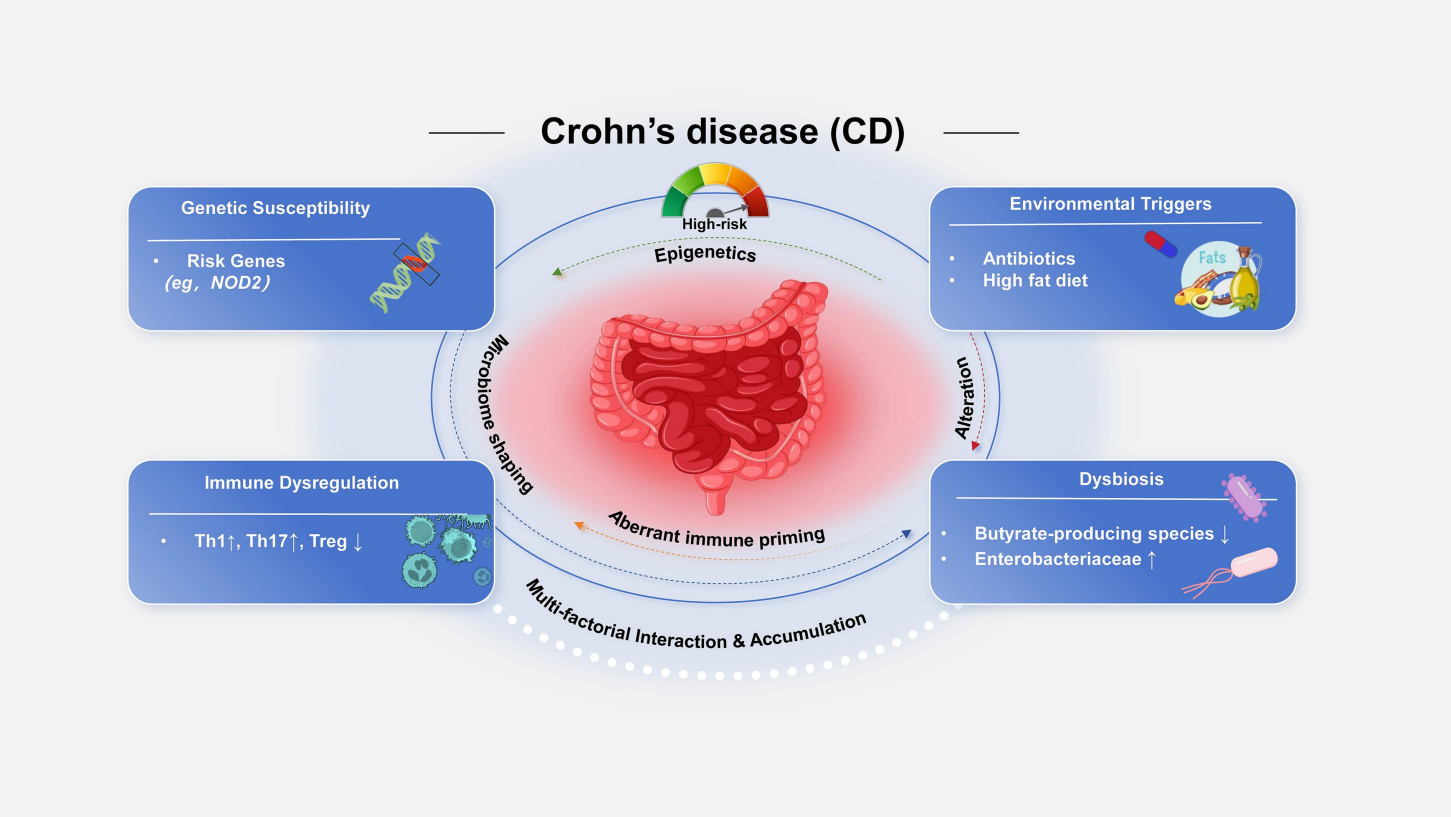
Key contributing factors in Crohn’s disease. The pathogenesis of Crohn’s disease (CD) is driven by the interplay of four major factors: genetic susceptibility, gut dysbiosis, immune dysregulation, and environmental triggers. Genetic risk variants, such as *NOD2*, increase disease susceptibility. Dysbiosis is characterized by reduced abundance of butyrate-producing bacteria and overgrowth of pro-inflammatory *Enterobacteriaceae*. Immune dysregulation is marked by an increased proportion of Th1/Th17 cells and a decreased proportion of regulatory T (Treg) cells. Environmental factors also contribute importantly to CD pathogenesis. Moreover, these four factors interact with and influence each other. Intestinal schematic designed by brgfx-Magnific.com (Free for commercial use WITH ATTRIBUTION license).

Crohn’s disease (CD), characterized by chronic and relapsing intestinal inflammation, is one of two predominant phenotypes in inflammatory bowel disease (IBD). The pathogenesis of CD involves dysregulation of the immune response to commensal gut pro-inflammatory microbiota in genetically susceptible individuals (Figure 1). However, the exact mechanisms underlying this dysregulation remain unclear. While there are some reviews focus on the role of Intestinal microbiota in IBD (Satokari, 2015), the mechanisms of microbe-host interaction (Butto et al., 2015; Lavelle and Sokol, 2020), and the clinical implications of the relationship between the microbiota and IBD (Sheehan and Shanahan, 2017), etc., the effect of host risk genes in CD mediated by pro-inflammatory microbiota is still need to be discussed.

An aberrant immune response has been proposed as the primary factor in the pathogenesis of CD. Pro-inflammatory T-helper cells (Th1 and Th17) are highly expressed in the intestines of patients with CD (Maloy and Powrie, 2011). Additionally, CD patients show increased secretion of tumor necrosis factor-α, interferon-γ, interleukin-17 (IL-17), and IL-23 (Fujino et al., 2003; Fuss et al., 2006; Wolk et al., 2007; Chao et al., 2014), which disrupts the balance of the regulatory/effector CD4^+^ T-cell axis in immune homeostasis. Another important factor involved in the development of CD is the intestinal microbiome. Mouse models were shown to spontaneously develop colitis in conventional environments, while germ-free mice had no intestinal inflammation (Sellon et al., 1998; Stepankova et al., 2007; Sheikh et al., 2024). However, introducing dysbiotic microbiota into wild-type (WT) germ-free mice increased their susceptibility to colitis (Lamas et al., 2016; Chen et al., 2026). Research into the underlying mechanisms of IBD revealed that the intestinal microbiota is determined partially by host genetics and also by immune homeostasis. For instance, genetic variations in immune function and mucosal barrier were found to be associated with microbial composition, which is an important factor in host phenotype determination. The purpose of this review is to elucidate the pathogenesis of CD caused by the effect of microbiota on mediating genetic factors.

## 2 Microbial features associated with the occurrence of CD

Intestinal microbial compositions vary among adults (Eckburg et al., 2005; Qin et al., 2010); however, a metagenome study identified similar metabolic features of these microbiotas among healthy subjects (Human Microbiome Project Consortium, 2012), with a level of taxa abundance shown to be similar at different taxonomic levels between different cohorts of healthy subjects (Turpin et al., 2016; Wang et al., 2016).

CD patients with dysbiosis share some common characteristics, including a reduced microbial alpha-diversity, which represents species richness and evenness (Andoh et al., 2012; Sha et al., 2013). Interestingly, the level of diversity negatively correlated with disease activities, demonstrating that lower diversity was associated with a higher activity index (Papa et al., 2012; Nylund et al., 2015). With the exception of the alpha-diversity of microbiota, CD patients also have significantly distinct microbial structures compared with healthy controls (Sabino et al., 2016; Sokol et al., 2017) as determined by principle coordinates analysis, which compares the variation of community structures among different subjects. This showed that clusters of healthy subjects were significantly different from clusters of CD patients. At the taxonomic level, characteristic alterations of microbiota composition revealed a significantly lower abundance of the phylum Firmicutes which normally dominates, especially Clostridia, and an overabundance of Proteobacteria, especially Enterobacteriaceae (Kaakoush et al., 2012; Imhann et al., 2018). Notably, the reduced abundance of multiple butyrate-producing bacteria species could explain the microbial features of CD patients (Takahashi et al., 2016).

As well as determining changes in microbiota, the metagenomic analysis of microbiota in CD patients has also been conducted to investigate the pathogenesis of CD. The findings indicated that basic metabolism and short-chain fatty acid (SCFA) production of the microbiota is decreased, and is accompanied by an increase in oxidative stress pathways (Morgan et al., 2012). The analysis of microbiota in CD patients also revealed an impaired ability of deconjugation, transformation, and desulfation to bile acids, which contributes to the observed reduction in secondary bile acids (SBA) (Duboc et al., 2013). SBA metabolism was reported to generate anti-inflammatory effects in the epithelial cells of the gut (Duboc et al., 2013; Biagioli et al., 2026).

## 3 Pro-inflammatory gut microbiota in CD patients

In CD patients, the decreased abundance of the butyrate-producing genera *Faecalibacterium*, *Ruminococcus*, *and Bifidobacterium* was observed, in the meantime, the butyrate metabolism was also decreased in the functional analysis (Furusawa et al., 2013; Velasquez-Manoff, 2015; Borren et al., 2021). In addition, the phylum Proteobacteria and the family Enterobacteriaceae were increased in CD patients, together with an increased endotoxin metabolism and enhanced intestinal inflammation. The observed alterations of the microbiota and corresponding metabolites in CD patients suggest an increased pro-inflammatory environment as well as a reduction in anti-inflammatory mediators (Viladomiu et al., 2022).

## 4 Dysbiosis in the development of CD

Gut microbiota plays a vital role in the pathogenesis of CD, and also modulates the immune system. Indeed, some species of Clostridia have been shown to induce colonic regulatory CD4^+^ T cells (Treg cells) and anti-inflammatory IL-10 to suppress the inflammatory response (Atarashi et al., 2013). CD remission is associated with the improvement of dysbiosis (Bertin et al., 2026), and several studies have reported correlations between members of the microbiota and the severity level of CD. For example, numbers of γ-Proteobacteria positively correlate with the Harvey–Bradshaw index (Guinet-Charpentier et al., 2017).

The microbiota composition is associated with various host phenotypes, including metabolic and immunological phenotypes. In recent studies, the correlation between microbiota composition and immune conditions has been investigated in animal models (Sheikh et al., 2024). A mouse model lacking Tata Element Modulatory Factor (TMF^−/−^ mice), which can produce thick and uniform colonic mucus, has a microbiota composition containing higher numbers of Firmicutes with increased diversity that demonstrate a decreased susceptibility to dextran sulfate sodium (DSS)-induced colitis compared with WT mice; importantly, when TMF^−/−^ mice were cohoused with WT mice, the latter developed a microbiota phenotype resistant to DSS-induced colitis, while TMF^−/−^ mice demonstrated enhanced susceptibility to DSS-induced colitis accompanied by a reduced abundance of Firmicutes (Bel et al., 2014). These findings suggest that microbiotas play an important role in host inflammatory reactions. In line with this, antibiotic treatment significantly reduced the level of ileitis in a CD-like mouse model, accompanied by alterations in the microbiota composition; however, restoration of the microbiota composition to pretreatment conditions promoted the severity of ileal inflammation (Schaubeck et al., 2016). The transfer of dysbiotic microbiota from ileitis conditions rather than the microbiota from healthy individuals can reproduce ileal inflammation (Schaubeck et al., 2016). Notably, Palm et al. devised a method to identify the colitogenic bacteria within the gut microbiota of IBD patients, which is highly coated by high-affinity IgA. Transfer of highly IgA-coated bacteria to germ-free mice dramatically increased susceptibility to colitis compared with low IgA-coated bacterial colonization, which implied that these bacteria have pathogenic properties (Palm et al., 2014). Taken together, dysbiotic microbiota appears to play a role in the pathogenesis of CD.

## 5 Risk genes contributing to the development of CD

Genome-wide association studies (GWAS) have identified more than 200 genetic susceptibility loci associated with IBD. Among the risk genes, *NOD2, CARD9, ATG16L1, IRGM*, and *FUT2* are well recognized. For instance, in a study on early-onset IBD, *NOD2* and *IRGM* variants were shown to increase the risk for CD (odds ratios (ORs) = 6.56 and OR = 2.32, respectively) (Pranculiene et al., 2016), while *NOD2* susceptibility loci can also predict the severity of CD (Schnitzler et al., 2014). These loci account for a modest variance of IBD. Another feature of these risk genes is that some are shared across different populations (Tremelling et al., 2008; Arimura et al., 2014), while others are population-specific (Inoue et al., 2002; Guo et al., 2004; Mahurkar et al., 2011). Rare variants with a high effect size on IBD susceptibility have also been found, including *Foxp3, IL-10R*, and *NDP52* (Moran et al., 2013; Okou et al., 2014; Inoue et al., 2022). Furthermore, the combined risk score, which includes a number of susceptible loci, was found to be predictive of disease severity (Haritunians et al., 2010). Interestingly, the risk score also indicated associations with other features of IBD, such as intestinal microbiota (Jacobs et al., 2022).

## 6 The role of host genetics in influencing the gut microbial composition

Compelling evidence for host genetic control of the gut microbiome comes from the twin study by Goodrich et al., who showed that monozygotic twins harbor more similar gut microbiota than dizygotic twins. They further identified multiple heritable microbial taxa, with the family Christensenellaceae displaying the strongest heritability (Goodrich et al., 2014). Skin microbiota has also been investigated in a twin study, revealing that monozygotic twins are most similar in terms of microbiota compared with dizygotic twins, mother-twins, and unrelated subjects (Si et al., 2015).

Genetic associations with bacteria of different taxonomic abundance have been increasingly investigated by GWAS (Kurilshikov et al., 2021; Lopera-Maya et al., 2022). Genetic risk scores were established for 11 functional genetic variants associated with IBD, including *NOD2, CARD9, ATG16L1, IRGM*, and *FUT2*. In healthy individuals, a high genetic risk for IBD was associated with a decrease in the relative abundance of the *Roseburia* genus (Imhann et al., 2018). Wang et al. performed GWAS analysis on gut microbiota in a combined cohort and observed an association of individual loci with either the abundance of particular bacterial taxa or variations in the microbial community structure (approximately 10% of the variation was explained by 42 loci) (Wang et al., 2016). In a study of a large healthy cohort of 1,098 individuals, an association was observed between 58 single nucleotide polymorphisms and the relative abundance of 33 taxa, including genes within or near identified loci involved in CD. Among these, four loci were replicated in a second replication cohort of 463 individuals, emphasizing the genetic effect on the composition of fecal microbes (Turpin et al., 2016). The role of host genetics in influencing the gut microbial composition was also investigated in the Hutterites. GWAS demonstrated a significant association of the relative abundance of at least eight bacterial taxa in each season with host genetic variants (winter: 15; summer: 14; combined: 8), including genera *Akkermansia* and *Lactococcus* (Davenport et al., 2015).

Microbiota heritability studies have not only been conducted in humans, but also in mouse and plant models. For example, heritable variation in total bacterial diversity has been found in the maize rhizosphere microbiome (Peiffer et al., 2013). Additionally, Benson and colleagues performed quantitative trait loci (QTL) analysis of microbiota in advanced intercrossed mouse lines, and identified 18 QTLs that were associated with various bacterial taxa in gut microbiota (Benson et al., 2010). Importantly, the relationship between the abundance of some bacterial taxa, corresponding QTL, and susceptibility to different diseases could be combined, demonstrating the potential role of microbiota in mediating the effect of the host genotype (Benson et al., 2010). The gut microbial composition was also shown to have important quantitative differences among BXD mouse strains, which were influenced by host genetics, while QTL mapping uncovered an association between QTL and particular taxa. For example, specific associations were detected between a QTL located on chromosome 4 and *Bacteroides*, a QTL on chromosome 15 and Rikenellaceae, and a QTL on chromosome 12 and Provotellaceae (McKnite et al., 2012). The above studies indicate that host genetics can influence microbial composition.

## 7 The interaction between pro-inflammatory gut microbiota and genetically susceptible host

The relationship between host genetics, gut microbiota, and host phenotypes remains unclear in humans. However, the finding of an association between a taxon known to affect obesity, and a variant, with body mass index (Davenport et al., 2015) aroused our interest in whether bacteria play a role in mediating the relationship between host genotypes and phenotypes. Dobson et al. found that microbiota-dependent nutrition is determined by host genetics; this was validated by loss-of-function mutations in identified genes that alter the microbiota-dependent nutritional patterns of *Drosophila melanogaster* (Dobson et al., 2015). Org et al. investigated the heritability of the microbiota composition in a panel of 110 diverse inbred strains of mice, and found that genetics could determine the abundance of the most common microbiota in a controlled environment. Meanwhile, the mouse response to diet, which was investigated by the cross-fostering strategy, was attributed to the microbial composition (Org et al., 2015). Chaston and colleagues studied the effect of the interaction of genetics and the microbiota on the nutritional indices of *Drosophila*, and demonstrated that the microbial composition is determined by genotypes, and that the microbiota could mediate the relationship between genotype and phenotype in response to changes in nutrition (Chaston et al., 2016). Together, these findings suggest that the microbiota, which is partially shaped by the host genome, can impact on host phenotypes, emphasizing the importance of the microbiota and genetic interaction.

Importantly, in healthy individuals, the genetic risk scores based on 11 functional genetic variants in *NOD2, CARD9, ATG16L1, IRGM*, and *FUT2* were inversely correlated with the relative abundance of *Roseburia*, a butyrate-producing genus (Imhann et al., 2018). A decrease in *Roseburia* abundance is characteristic of dysbiosis in IBD patients (Gevers et al., 2014). Interestingly, healthy siblings of CD patients with an elevated risk of IBD also demonstrated a decreased abundance of *Roseburia* (Hedin et al., 2014). Butyrate, which can be the primary energy source for colonocytes, was shown to increase the expression of *MUC* genes in goblet cells, resulting in the increased secretion of mucin (Gaudier et al., 2004; Donohoe et al., 2011). Mucin is the major element of intestinal tract mucus, which spatially separates the epithelium from dietary and microbial antigens, thus contributing to immune homeostasis. Butyrate also promotes an immunological balance through promoting the secretion of IL-18 in epithelial cells, increasing colonic Treg cells, and inducing the production of anti-inflammatory IL-10. In line with this, butyrate treatment in IBD improved disease activity (Scheppach et al., 1992). Accordingly, an association of the *NOD2* risk allele count with the increased relative abundance of Enterobacteriaceae was documented in IBD patients (Knights et al., 2014). As expected, *NOD2*-deficient mice demonstrated dysbiosis in their intestinal microbiota, which increased the susceptibility to colitis in WT cage-mates after transferring the dysbiotic microbiota (Couturier-Maillard et al., 2013). A similar observation was also made in another mouse model with spontaneous intestinal inflammation (Garrett et al., 2010). The microbial community was compared between patients with CD, low-risk healthy controls, and healthy siblings of CD patients with an elevated risk of developing CD. The results revealed that the healthy siblings exhibited a dysbiotic mucosal microbiota characterized by a lower diversity of core microbiota and lower abundance of *Faecalibacterium prausnitzii* compared with healthy controls (Hedin et al., 2016). Notably, *F. prausnitzii* is also involved in the production of SCFAs (including butyrate) and contributes to gut homeostasis through a SCFA-dependent and SCFA-independent pathway (Benus et al., 2010; Huang et al., 2016; Rossi et al., 2016; Baradaran Ghavami et al., 2021).

**Figure 2:**
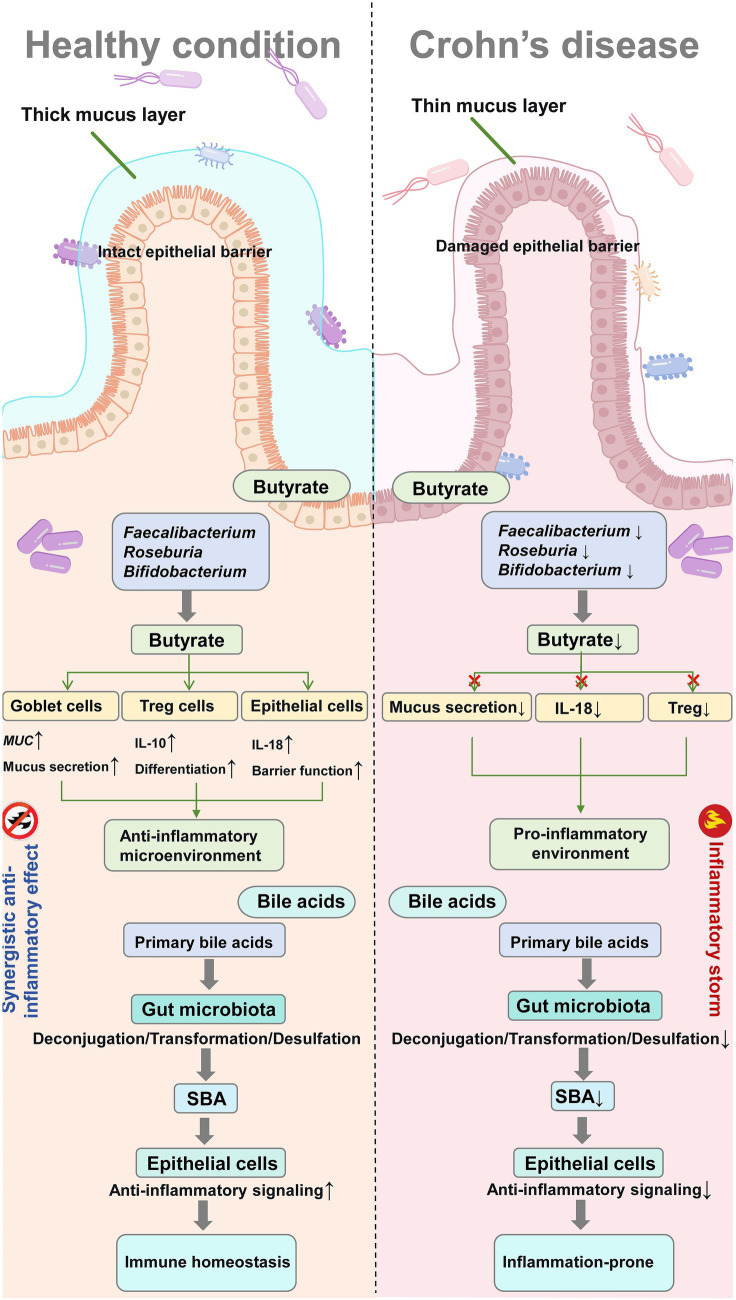
Schematic comparison of the gut in health and Crohn’s disease. In the healthy state (left), beneficial bacteria (e.g., *Faecalibacterium, Bifidobacterium*) produce butyrate, which strengthens the epithelial barrier and induces regulatory T (Treg) cell differentiation, establishing an anti-inflammatory microenvironment. Meanwhile, microbiota-mediated conversion of primary to secondary bile acids (SBA) supports immune homeostasis. In Crohn’s disease (right), depletion of butyrate-producing bacteria and impaired bile acid metabolism disrupt barrier integrity and Treg differentiation, collectively promoting an inflammation-prone state.

These associations imply that the pro-inflammatory composition of the microbiota in genetically susceptible individuals could play a fundamental role in IBD pathogenesis (Figure 2).

## 8 Conclusion and future perspectives

In summary, the pathogenesis of Crohn’s disease is intimately driven by the interplay between host genetics and the gut microbiota. GWAS-identified risk variants, including *NOD2*, *ATG16L1*, *CARD9*, *IRGM*, and *FUT2*, predispose individuals to a pro-inflammatory microbiota characterized by a depletion of butyrate-producing bacteria (e.g., *Faecalibacterium*, *Roseburia*) and an expansion of Enterobacteriaceae. This dysbiotic microbiota, in turn, impairs intestinal barrier integrity, disrupts bile acid metabolism, and fuels immune dysregulation, supporting the model that pro-inflammatory gut microbiota mediates the genetic risk of CD.

Future large-scale, multi-ethnic GWAS integrating host genomes and gut metagenomes are needed to refine the genetic architecture of microbial composition and pinpoint causal taxa. In parallel, longitudinal multi-omics studies should dissect how host genotype, microbiota, metabolites, and environmental exposures dynamically interact to shape disease onset, severity, and progression. Clinically, integrating genetic risk scores with microbiota profiling could identify individuals at high risk for early-onset, severe, or recurrent CD, enabling early preventive strategies. Microbiome-targeted strategies are particularly appealing in genetically susceptible hosts: supplementation with next-generation probiotics, bacteriophage therapy targeting pathobionts, dietary interventions designed to enhance short-chain fatty acid production, and fecal microbiota transplantation from healthy donors all represent promising avenues to normalize microbial composition and metabolic function (Wang et al., 2026). Ultimately, these integrative strategies aim to shift CD management from reactive immunosuppression toward proactive, genotype-guided, microbiome-informed precision medicine.
